# Sphingosine-1-Phosphate and Macrophage Biology—How the Sphinx Tames the Big Eater

**DOI:** 10.3389/fimmu.2019.01706

**Published:** 2019-07-19

**Authors:** Andreas Weigert, Catherine Olesch, Bernhard Brüne

**Affiliations:** ^1^Faculty of Medicine, Institute of Biochemistry I, Goethe-University Frankfurt, Frankfurt, Germany; ^2^German Cancer Consortium (DKTK), Partner Site Frankfurt, Frankfurt, Germany; ^3^Project Group Translational Medicine and Pharmacology TMP, Fraunhofer Institute for Molecular Biology and Applied Ecology, Frankfurt, Germany; ^4^Frankfurt Cancer Institute, Goethe-University Frankfurt, Frankfurt, Germany

**Keywords:** sphingosine-1-phosphate, macrophages, macrophage polarization, cancer, atherosclerosis, infection, inflammation

## Abstract

The sphingolipid sphingosine-1-phosphate (S1P) is produced by sphingosine kinases to either signal through intracellular targets or to activate a family of specific G-protein-coupled receptors (S1PR). S1P levels are usually low in peripheral tissues compared to the vasculature, forming a gradient that mediates lymphocyte trafficking. However, S1P levels rise during inflammation in peripheral tissues, thereby affecting resident or recruited immune cells, including macrophages. As macrophages orchestrate initiation and resolution of inflammation, the sphingosine kinase/S1P/S1P-receptor axis emerges as an important determinant of macrophage function in the pathogenesis of inflammatory diseases such as cancer, atherosclerosis, and infection. In this review, we therefore summarize the current knowledge how S1P affects macrophage biology.

## Introduction

In 1887, Metchnikoff published work on the nature host cells combating bacterial infection. He suggested the large cells he and other before him had observed taking up whole cells or cell fragments, to be named macrophages, as opposed to microphages (polymorphonuclear leukocytes) who specialized in combating bacteria (1). The term macrophage consequently is a composite of the Greek words *makros*, meaning large, and *phagein*, to eat, and denotes big eating cells. Today we are aware that macrophages are more than just big eaters, playing a multitude of crucial roles in development and maintaining adult tissue homeostasis. To be able to fulfill these roles, they command an enormous sensory repertoire to recognize cues suggesting endangered or disturbed homeostasis. One of these cues is the sphingolipid S1P that is produced during inflammation and upon tissue damage. Sphingolipids were named after the Sphinx by J. L. W. Thudichum in 1884 due to the enigmatic biochemical properties of their common backbone, the alcohol sphingosine. The Sphinx in Greek mythology poses riddles to travelers and kills them if they fail to answer correctly, whereas in Egyptian mythology the Sphinx is a rather benevolent guardian of sacred sites. Like the enigmatic Sphinx, S1P affects macrophage biology in different, sometimes antithetical ways. Here, we review the interaction of the sphinx and the big eater. We start with an introduction of the protagonists and their role in inflammation and tissue homeostasis. Next, we summarize the current knowledge on molecular mechanisms how S1P attracts macrophages and determines their survival, followed by reviewing how S1P affects the signature functions of macrophages, i.e., phagocytosis and the regulation of inflammation. Finally, we discuss how these S1P-dependent mechanisms affect macrophage function in pathological settings.

## More Than Big Eaters—Macrophage Function in Homeostasis and Disease

Macrophages are ubiquitous, yet diverse tissue-resident immune cells, involved in maintaining tissue integrity and function. They sense and actively respond to disturbances in tissue homeostasis by initiating, but also resolving inflammation (2, 3). The diverse functions of macrophages are tissues-specific and range from basic tasks, such as rearranging the extracellular matrix and taking up and recycling cellular and molecular debris, to highly specialized functions such as controlling tissue innervation or promoting conductance in the heart by modulating electrical properties of cardiomyocytes (3–6). Upon tissue injury, macrophages recognize new molecular patterns from dead cells or invading microorganisms. In turn, this mounts an immune response, e.g., by recruiting new inflammatory cells to the site of tissue disturbance. Once a noxa is cleared with the help of resident and recruited macrophages, macrophages participate in removing (dead) inflammatory cells by phagocytosis. At the same time they contribute to restore the tissue by promoting angiogenesis and reparative signaling in stroma and parenchyma (7–10). Their ability to cope with the changing demands during an acute inflammatory reaction suggests a remarkable plasticity.

Macrophage function is, to a large extent, dictated by the dominating microenvironment, rather than genetic imprinting. In tissues, macrophages have different developmental origins (11–13). They can be derived from early hematopoiesis in the yolk sac (11–17) or the fetal liver, without transitioning through a monocytic intermediate stage (16, 17). Post-natally, macrophages may derive from hematopoietic stem cell-derived monocytes from the bone marrow (18, 19). Tissue-resident macrophages of embryonic origin often self-renew by *in-situ* proliferation, whereas monocyte-derived macrophages are frequently, but not always, short-lived and continuously replaced (11–14, 20). During depletion of the resident macrophage pool upon e.g., inflammation or experimental means, monocytes or other macrophage progenitors readily integrate into the tissue macrophage pool and become self-renewing cells (11, 12, 14, 20, 21). Moreover, transplantation of mature macrophages between tissues alters their transcriptional program to fit the recipient tissue macrophage pool (20). Finally, distinct macrophage subsets, partly of similar developmental origin, are found in specialized niches within one tissue (22–25).

The notion that the microenvironment determines macrophage function is further supported by identifying tissue-specific transcription factors that are required to establish tissue macrophage identity. All macrophages depend on the lineage-determining transcription factor PU.1, whose expression is triggered by colony stimulating factor-1 (CSF1) or interleukin (IL-)34 (IL-34), which signal through colony stimulating factor-1 receptor (26, 27). Moreover, the transcription factor ZEB2 is required for macrophage identity across a number of tissues (28). On top of this basic transcriptional program, tissue-specific transcriptional regulators were identified that are activated downstream of tissue-enriched molecular cues. These include SPI-C in progenitors of red pulp and bone marrow macrophages (29), GATA6 in peritoneal macrophages (30, 31), peroxisome proliferator-activated receptor (PPAR) in alveolar macrophages (32), SMAD transcription factors and myocyte-specific enhancer factor 2c (MEF2c) in microglia (33, 34), liver x receptor (LXRα) in Kupffer cells, as well as Runt-related transcription factor 3 (RUNX3) in intestinal macrophages and Langerhans cells (20, 35).

Despite these lineage- and tissue-imprinted molecular programs, macrophages retain a remarkable degree of plasticity to respond to the appearance of new molecular cues indicative of a disturbed homeostasis. These cues are sensed by a repertoire of receptors on macrophages and activate transcriptional enhancers or repressors, generating a large number of possible activation states (36–39). A considerable body of research aimed at defining discrete macrophage polarization states *in vitro* by using sets of specific molecular or functional markers, which are thought to serve as predictors of macrophage function in living organisms (36, 40–42). Frequently used is the M1/M2 nomenclature, where M1 macrophages are stimulated with interferon-γ (IFN-γ) and lipopolysaccharide (LPS) to mimic a condition arising during type 1 inflammation (defense against microbial infection), whereas M2 macrophages are stimulated with the TH2 cytokines IL-4 or IL-13 to mimic conditions of type 2 inflammation (helminth infection). Specific marker signatures have been assigned to these cells. M1 macrophage activation creates an anti-microbial, pro-inflammatory cell with a transcriptional signature defined by activation of nuclear factor kappa-light-chain-enhancer of activated B cells (NF-κB), signal transducer and activator of transcription 1 (STAT1) and interferon regulatory factor 5 (IRF 5) (43, 44). M1 or classically activated macrophages produce pro-inflammatory mediators such as tumor necrosis factor-α (TNF-α), IL-1β, IL-6, and IL-12, generate reactive oxygen and nitrogen species (ROS/RNS), and activate T cells to produce type 1 cytokines. M2 or alternatively activated macrophages display activation of the transcription factors STAT6 and IRF4 to express specific chemokines including CCL17 and CCL18, phagocytic receptors such as the mannose receptor CD206, arginases (ARG1/2) to limit NO production, and mediators that modulate the extracellular matrix. All of them are originally induced to combat extracellular parasites (36, 39, 44–46). The M1/M2 nomenclature is useful, although naturally limited since macrophages in a tissue never face M1 or M2-specific stimuli. In fact, IL-4 and IFN-γ are often produced simultaneously during inflammation (47). In analogy to T cells, IL-4, and IFN-γ, besides inducing discreet transcriptional outputs, mutually suppress the impact of the corresponding signaling pathway to affect the macrophage phenotype. IL-4 suppresses enhancer regions in a large set of inflammatory genes directly via STAT6 (48), while IFN-γ induces a loss of enhancer binding by the transcription factor MAF in M2 genes to reduce chromatin accessibility (49). Even when supplied together in cell culture, IFN-γ and IL-4 mutually inhibited epigenomic and transcriptional changes induced by each cytokine alone, while allowing the expression of core functional parameters such as IFN-γ-triggered antiviral genes (47). Moreover, macrophage polarization by IFN-γ or IL-4 appears to be a transient rather than a stable process (47, 50, 51), which makes sense when different functional needs arise during the course of an inflammatory reaction. Therefore, “pure” M1 or M2 macrophages will not be found in a complex environment, and claiming distinct functions from a small set of markers expressed by macrophages in a tissue needs to be approached carefully. Nevertheless, understanding mechanisms that regulate macrophage plasticity is of importance, since dysregulation of macrophage activity is connected to human pathologies including major causes of premature death such as infection, atherosclerosis, fibrotic diseases, and cancer (3, 37).

## Enter: the Sphinx—S1P and Its Receptors in Immunity

Sphingosine-1-phosphate (S1P) is a biologically active lipid mediator being produced in and affecting macrophages. With central roles of macrophages during inflammation and cancer the sphere of S1P actions touches ground under a number of physiological as well as pathological settings. Production, degradation, and biological actions of S1P in the mammalian system have been reviewed in depth (52–58) and are only briefly recapitulated. Central to sphingolipid metabolism is ceramide, which is a hub for sphingolipid synthesis and degradation (59). *De novo* synthesis of ceramide starts by condensation of serine and palmitoyl-CoA to form 3-keto-dihydrosphingosine, which is subsequently reduced to dihydrosphingosine and N-acylated to form a large group of dihydroceramides (60). A desaturase then produces corresponding ceramides. Ceramides can either be phosphorylated, or glycosylated to form glucosylceramides, which are processed and exposed at the plasma membrane as glycosphingolipids. Alternatively, ceramides can be converted to sphingomyelin, also being incorporated into the outer cell membrane. There, sphingomyelin can be attacked by neutral or acidic sphingomyelinase and converted back to ceramide. In turn, ceramides are cleaved by ceramidases to generate sphingosine, which gets phosphorylated by sphingosine kinase-1 (SPHK1) or−2 (SPHK2) to form S1P (55, 61). S1P can be transformed in the salvage pathway via sphingosine back to ceramide or irreversibly degraded by S1P lyase (SGPL1) to hexadecenal and phosphoethanolamine. S1P, produced at the plasma membrane, can be exported from cells by ATP-binding cassette (ABC) transporters or spinster 2 (Spns2) (62, 63). Once outside cells, S1P is recognized by a family of five distinct G-protein coupled receptors (S1PR1-5) that initiate autocrine, “inside-out,” or paracrine signaling (64). Cell type-selective expression of distinct S1P receptors and their coupling to different G-alpha subunits allows S1P to exert a multitude of signaling qualities (53). S1P can also signal intracellularly via several less generalized and commonly accepted targets (65–67), which also plays a role during macrophage activation.

Macrophages express all five S1P receptors, albeit receptor expression varies among different macrophage subtypes and seems related to distinct functional properties (67). S1P receptors belong to a family of seven helix transmembrane G-protein coupled receptors (GPCR), linked to either G_i_, G_q_, and/or G_12/13_. Concomitantly, directly tied signaling pathways including small GTPases, phospholipases, PI3K, or adenylyl cyclase are affected, which in turn initiate a myriad of diverse signals. Specific G-protein-coupled receptors (S1PR) signaling is commonly connected to cell migration, proliferation, and differentiation, with individual S1P receptors partly mediating convergent and partly mediating antithetic responses (68). For instance, S1PR1, coupling to G_i_ promotes the migration of lymphocytes, while S1PR2, which couples to G_i_, G_q_, and G_12/13_, restricts migration (69). This reciprocal interaction regulates among others B cell localization in lymphatic organs. On the other hand, S1PR1/2 and 3, the latter also coupling to G_i_, G_q_, and G_12/13_, appear to jointly coordinate vascular development during embryogenesis in mice and zebrafish (70, 71).

**Figure 1:**
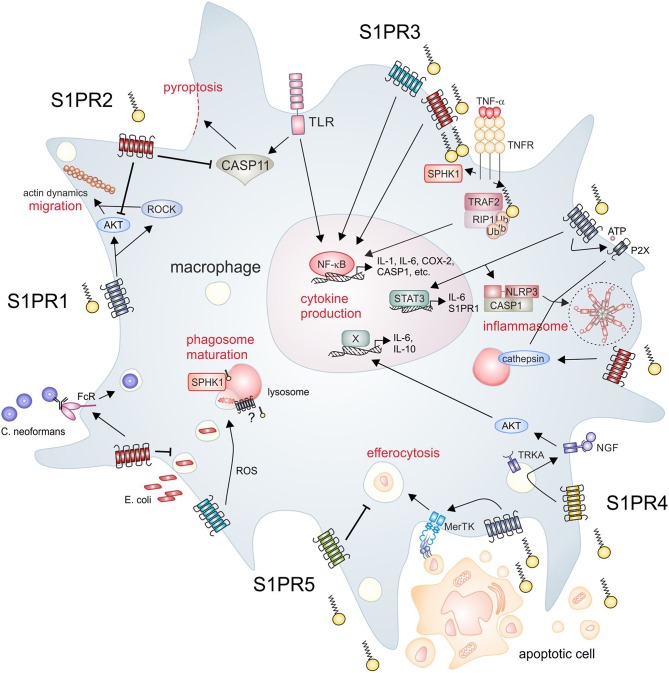
S1P signaling and macrophage function. The SPHK/S1P/S1PR axis regulates essential macrophage functions. Clockwise from upper left, S1PR1 signaling promotes macrophage migration, which is inhibited by S1PR2. Moreover, S1PR2 inhibits endotoxin-induced pyroptosis. Intracellular S1P produced by SPHK1 activates inflammatory pathways in macrophages, which are also activated downstream of S1PR2/3. S1PR1 mainly signals through STAT3 to induce cytokine production, but also induces expression of NLRP3. S1PR1 and S1PR2 are furthermore involved in the activation of the NLRP3 inflammasome trough divergent mechanisms. S1PR2 signaling promotes the opsonin-dependent uptake of the pathogenic fungus C. neoformans, but blocks uptake of bacteria such as E. coli. S1PR3 promotes bacterial killing via promoting ROS generation. SPHK1 is required for phagosome maturation, although it is unclear if S1PR signaling is involved in this process. Apoptotic cells release S1P to activate signaling through S1PR1, which promotes efferocytosis. In turn, S1PR5 blocks efferocytosis. S1PR4 is activated by apoptotic cell derived S1P to promote cytokine expression dependent on TRKA shuttling. AKT, protein kinase B; CASP, caspase; COX-2, cyclooxygenase-2; FcR, Fc receptor; IL, interleukin; MerTK, proto-oncogene tyrosine-protein kinase MER; NF-κB, nuclear factor “kappa-light-chain-enhancer” of activated B-cells; NGF, nerve growth factor; NLRP3, NACHT, LRR, and PYD domains-containing protein 3; P2X, P2X purinoceptor; RIP, receptor interacting protein; ROCK, rho-associated, coiled-coil-containing protein kinase; ROS, reactive oxygen species; S1P, sphingosine-1-phosphate; S1PR, sphingosine-1-phosphate receptor; SPHK, sphingosine kinase; STAT, signal transducer and activator of transcription; TLR, toll-like receptor; TNF, tumor necrosis factor; TNFR, TNF receptor; TRAF, TNF receptor-associated factor; TRKA, tropomyosin receptor kinase A.

S1P is a prototypical molecular signal that is induced upon disturbance of tissue homeostasis. Normally, its levels in tissues are too low to activate specific receptors, with the exception of the circulation, where S1P levels reach nano- to micromolar concentrations (72). However, during inflammation S1P levels rise and are sensed, among others cells by macrophages. As macrophages are exposed to multiple signals from their environment, which allows them to adjust their output repertoire under homeostatic, inflamed, or regenerative conditions, S1P production, S1P receptor expression and/or signaling might add to the complexity of their functional properties. On the following pages we therefore summarize the impact of the S1P signaling system on macrophage responses (summarized in Figure 1), and discuss if modulation of this system might be therapeutically attractive.

## Attracting the Eater—S1P and Macrophage Homeostasis

In the last years it became evident that S1P plays an important role in tissue surveillance by recruiting immune cells and modulating their life-span (65). Consequently, S1P affects macrophage-driven tissue homeostasis by, among others, mediating macrophage differentiation, migration and survival. S1P, as shown for various other cell types, also serves in macrophages as an anti-apoptotic signal. It is suggested that S1P prevents caspase-induced apoptosis of macrophages by inducing the expression of anti-apoptotic proteins such as B-cell lymphoma 2 (Bcl-2) and B-cell lymphoma extra-large (Bcl-XL) through the activation of phosphoinositide-3-kinase (PI3K), extracellular-signal regulated kinase (ERK)1/2 and Ca^2+^ signaling pathways or changing the cellular balance of ceramide to sphingosine or S1P (58, 73, 74). A more recent study showed that S1P acted as an anti-apoptotic component of high-density lipoprotein (HDL) by inducing inhibitor of apoptosis (IAP) family member survivin via STAT3 in THP-1 and RAW264.7 macrophages (75). This was blocked by antagonists against S1PR2/3, indicating cooperative signaling by S1PR2/3 under these conditions (75). Reduced caspase-induced cell death of macrophages by S1P may extend beyond apoptosis. S1PR2 signaling was demonstrated to reduce caspase-11 protein expression in peritoneal macrophages, thereby limiting macrophage pyroptosis (76). Shifting the cellular balance of ceramide to sphingosine/S1P toward the latter seems not only to be important for macrophage survival but also plays a role during the differentiation of blood monocytes to macrophages. Monick et al. showed that during macrophage differentiation levels of acid ceramidase increased, which enhanced the lifespan of macrophages, most likely due to the conversion of ceramide to sphingosine, resulting in higher intracellular S1P levels (77).

For macrophages to maintain tissue homeostasis they not only need to regulate their number by survival, but they also have to be in the right place at the right time. Accordingly, S1P can also serve as a lipid attraction signal to guide macrophages to the sites of inflammation and tissue repair (78). It has been shown that S1P-dependent macrophage migration is strictly dependent on their S1PR profile. Thereby, S1PR1 signaling seems to be pro-migratory as shown for peritoneal macrophages involving Rho kinase and PI3K-Akt1 signaling and for bone marrow-derived macrophages (BMDM), stimulated by S1P-enriched extracellular vesicles during hepatic lipotoxicity (79, 80). S1PR1 is also essential for post-inflammatory macrophage emigration as shown in a mouse model of resolving peritoneal inflammation, harboring a macrophage-specific deletion of S1PR1, which reduced emigration of macrophages from the site of inflammation (81). More recently it has been discussed that S1PR4 signaling may inhibit the pro-migratory actions of S1PR1 and that the S1PR1/S1PR4 ratio is important for the emigration of pro-inflammatory M1 macrophages from sites of inflammation. It was demonstrated that M1 macrophages exhibit a higher S1PR1/S1PR4 ratio than M2 macrophages (82). However, further studies for example inhibiting S1PR1 and−4 individually in macrophages are needed to clarify the functional role of the S1PR4 to S1PR1 counter-regulation during inflammation. In contrast to the pro-migratory actions of S1PR1, S1PR2 signaling was proposed to inhibit macrophage trafficking by stimulating cAMP production, thereby attenuating Akt phosphorylation, as demonstrated using S1PR2 knockout mice in a peritonitis model of acute inflammation (83). These findings exemplified the antithetic properties of S1PR1 and S1PR2 in immune cell migration. In support of this concept, chemotaxis of osteoclast precursors, with osteoclasts being bone macrophages, is reciprocally regulated by S1PR1/2, which serves to fine-tune their localization in bones (84). However, Yang et al. proposed S1PR2/3 on BMDM to be pro-migratory, possibly due to the fact that both receptors share activation of similar signaling pathways involving PI3K and Ras-related C3 botulinum toxin substrate 1 (Rac1) (85). In the same study they excluded an involvement of S1PR1 in macrophage migration by stimulating BMDM with the selective S1PR1 inhibitor W146, which did not alter S1P-induced BMDM migration. This observation is rather contradictory to a number of studies discussed above, which clearly indicate a role for S1PR1 signaling in inducing macrophage migration. Macrophages may even switch their S1PR profile toward S1PR1 to allow migration to the lymphatics, similarly to dendritic cells (81, 86). Since macrophages are the most plastic cells of the immune system, contradictory studies on macrophage-specific S1P receptor functions are most likely due to different S1PR expression profiles, sources and distinct *in vitro* macrophage differentiation protocols. Further studies are required to delineate under which circumstances signaling through S1PR1 and S1PR2 cooperate or oppose each other during macrophage migration. In summary, in order to maintain tissue homeostasis, macrophages need to survive under stress conditions and need to migrate to specific tissue sites. For these actions the S1P/S1PR signaling axis appears to be critical as the above mentioned studies suggest.

## Preparing the Meal and Aiding in Digestion—Impact of S1P on Phagocytosis

Phagocytosis of pathogens or cellular debris is a key function of macrophages, as their name suggests. S1P has been shown to be involved in both, uptake of pathogens and cellular debris, i.e., dying cells. While these two processes occur via different molecular mechanisms, they both can be broken down into similar steps, i.e., recognition of the phagocytic material, phagosome formation, and phagosome content removal e.g., phagosome-lysosome fusion (87). S1P signaling participates in each of these three steps in macrophages.

S1P is produced by cells upon induction of apoptosis (88). It acts on macrophages to alter their functional phenotype, as outlined in the next paragraph. Moreover, S1P serves as a find-me signal to attract phagocytes to the dying cell for removal (78). This system can be hijacked by *Yersinia pestis*, which triggers cell death in macrophages. The resulting S1P release serves to recruit further macrophages, which are then infected to promote spread of bacteria (89). Besides altering macrophage activation and promoting recruitment, recent data suggest an involvement of S1P in priming macrophages for uptake of cell debris (90). Apoptotic cell-derived S1P triggered erythropoietin (EPO) signaling in murine macrophages, which induced upregulation of phagocytic receptors, including CD36 and Mer tyrosine kinase (MerTK) (90). Accordingly, mice lacking the EPO receptor in myeloid cells showed delayed clearance of apoptotic cells and lupus-like autoimmune symptoms. Interestingly, not only S1P produced by dying cells appears to be involved in apoptotic cell phagocytosis (efferocytosis). Inhibition of SPHKs in macrophages by pharmacological inhibitors or cigarette smoke reduced efferocytosis in human THP-1 macrophages. Addition of exogenous S1P or the S1PR agonist FTY720 reversed cigarette smoke-induced inhibition of efferocytosis and promoted macrophage SPHK functionality (91). Accordingly, the S1P transporter SPNS2 was decreased in smoke-exposed bronchial epithelial mice, correlating with reduced efferocytosis (92). Unexpectedly, smoke exposure increased SPNS2 expression in alveolar macrophages and upregulation of the S1PR5 in alveolar macrophages was associated with impaired efferocytosis (92–95). Thus, S1P receptors may differ in their capacity to promote or inhibit efferocytosis and the S1P system in both, macrophages and dying cells appears to be involved in chronic obstructive pulmonary disease (COPD), which is induced by smoking and linked to impaired efferocytosis.

It remains unclear if S1P only promotes efferocytosis by increasing phagocytic receptor expression, or whether S1P signaling may also participate in the actual engulfment machinery. So far, the picture is clearer when looking at pathogen uptake as S1P facilitates the expression of uptake receptors. The phagocytic receptor FcγRII (CD32) is induced in human macrophages (96). This may require signaling through S1PR2, since S1PR2-deficient macrophages expressed significantly lower levels of FcγRI, II, and III (97). Thereby, S1P triggered S1PR2-dependent phagocytosis of the pathogenic fungus *Cryptococcus neoformans* (*C. neoformans*) (97). Apparently, S1P promotes opsonin-dependent phagocytosis of pathogens. In contrast, S1PR2-deficient murine macrophages phagocytosed *Escherichia coli* (*E. coli*) more efficiently, which was attributed to reduced RhoA-dependent cell contraction, but increased formation of lamellipodial protrusions when S1PR2 was absent (98). Importantly, phagocytosis of *E. coli* occurred opsonin-independent, adding to the notion that S1P modulating effects on phagocytosis depend on the S1PR being triggered and the mechanism of uptake. *E. coli* phagocytosis relied on RhoA and therefore actin dynamics. Two earlier studies suggested that S1P modulates actin assembly to promote phagosome formation. S1P promoted an ADP to ATP conversion and subsequent purinergic ligand-gated ion channel P2X7 signaling in the phagosome lumen or extracellularly, both promoting actin assembly at the plasma membrane in murine macrophages (99, 100). Moreover, S1P triggered ATP release from RAW264.7 macrophages by activating volume-regulated anion channels downstream of S1PR1. This required the actin cytoskeleton, suggesting that S1PR1 signaling may affect the actin cytoskeleton to induce a feed-forward mechanism involving purinergic signaling to promote phagosome formation and maturation. A critical function for S1P in phagosome maturation is supported by the observation that exogenous S1P promotes the interaction of phagosomes with the actin cytoskeleton to allow trafficking of the phagosome toward lysosomes for lysosomal fusion (101). Phagolysosome generation in *Mycobacterium tuberculosis* (*M. tuberculosis*) infected human macrophages required exogenous S1P-dependent phospholipase D activity, thereby promoting killing of *M. tuberculosis* (102). Besides *M. tuberculosis*, exogenous S1P was also shown to enhance killing of other mycobacteria by macrophages (103). However, S1PR3 was implicated in promoting phagosome maturation in mouse macrophages, since S1PR3-deficient peritoneal macrophages treated with heat-killed *E. coli* showed reduced phagolysosome fusion (104). Besides exogenous S1P, intracellular S1P formation was required for killing of mycobacteria by macrophages. Targeting SPHK1 genetically or pharmacologically rendered murine RAW 264.7 macrophages sensitive to infection with *M. smegmatis*, whereas overexpression of SPHK1 promoted killing. This was accompanied by SPHK1-dependent expression of the late phagosome marker LAMP2, but also SPHK1-dependent NO formation (105). Strikingly, it was further shown that intracellular *M. tuberculosis* blocked phagosome maturation by impairing SPHK activity (106). *M. tuberculosis* is known to block its killing in fused acidic phagolysosomes. Ingestion of dead, but not living *M. tuberculosis* induced SPHK1 sphingosine kinase activity and translocation to nascent phagosomes in human macrophages, followed by an increase in intracellular Ca^2+^ (106, 107). SPHK1 translocation in itself was Ca^2+^ dependent as suggested by the use of an intracellular Ca^2+^ chelator. Besides mycobacteria, a role for SPHK1 in controlling *C. neoformans* infection was suggested (108). In particularly, SPHK1 restricted intracellular *C. neoformans* growth in alveolar macrophages and also restricted macrophage infection with *Leishmania donovani* (109), although it is unclear if this was dependent on phagolysosome formation.

Collectively these data indicate that intracellular as well as extracellular S1P, presumably via S1PR signaling, has microbicidal potential by modulating pathogen uptake, phagosome formation, maturation, and phagolysosome fusion. Moreover, S1PR3 activation on mouse macrophages promoted ROS formation and therefore killing of ingested bacteria (104). The notion of a crucial role of the S1P system in pathogen control is underscored by observations that microbial SGPL1 promotes their survival in macrophages. SPGL1 from *Burkholderia pseudomallei* was required for phagosome evasion, presumably by lowering S1P levels, as indicated by observations that S1P and S1PR1 agonists increased bacterial content in lysosomes and reduced their intracellular survival (110). Moreover, SGPL1 from *Legionella pneumophila* promoted intracellular pathogen survival by blocking autophagy through disrupting sphingolipid metabolism, thereby again preventing lysosomal killing of pathogens (111). Therefore, a number of different pathogens have developed strategies to target the S1P system and to evade intracellular degradation in lysosomes (106, 110, 111).

## Adding Flavor—S1P and Macrophage Polarization

During recent years it became apparent that the impact of S1P on the macrophage phenotype is not restricted to the M1/M2 paradigm. Rather, S1P modulates macrophage responses according to the local environment, the compartmentalization of S1P, i.e., intra vs. extracellular S1P, and the S1P receptors activated on cells. Consequently, S1P not only promotes the production of M2 but also of M1-associated macrophage markers.

Intracellular S1P produced by SPHK1, likely at the plasma membrane, was suggested as a cofactor involved in inflammatory macrophage activation. Inflammatory macrophage activation is triggered by microbial components such as LPS, with or without type 1 lymphocyte-derived IFN-γ. As a consequence, TNF-α is rapidly produced by mechanisms including proteolytic shedding from the plasma membrane (112), and increasing mRNA stability (113). TNF-α then binds to its cognate receptors to feed-forward promote canonical NF-κB activation, which requires TNF receptor-associated factor 2 (TRAF2) to polyubiquitinate receptor interacting protein 1 (RIP1). SPHK1 was shown to be activated downstream of TNF receptor activation and to physically interact with TRAF2. SPHK1-derived S1P then acted as cofactor for TRAF2, allowing polyubiquitination of RIP1 (114, 115). Also IL-1 signaling, another feed-forward NF-κB activator following microbial encounter, required SPHK1-dependent S1P as an intracellular cofactor (116). Besides TNF and IL-1β, SPHK1 is also rapidly activated downstream of other inducers of inflammatory macrophage activation, including LPS (117–119), and LPS in combination with palmitate (120), although it is unclear if SPHK1-derived S1P acts as a cofactor for intracellular LPS signaling. Stimulation of human THP-1 macrophages or mouse microglia with LPS required SPHK1 activity to produce IL-6, IL-1β, TNF-α, and/or NO (117, 118), whereas SPHK1 was dispensable for LPS, but not LPS/palmitate-induced IL-6 production, and TNF-α induced cyclooxygenase (COX)-2 expression in mouse RAW264.7 macrophages (119, 120). Accordingly, SPHK1-deficient mice showed decreased joint inflammation in a model of murine TNF-α-induced arthritis (121). In contrast, SPHK1-deficient mice were not protected from collagen-induced arthritis and thioglycollate-triggered peritonitis, indicating that SPHK1 activation may be restricted to specific inflammatory stimuli (122). It is important to note that mouse macrophages lacking both SPHK isoforms did not show any alterations in cytokine production *in vitro* and induction of LPS or thioglycollate-induced inflammation *in vivo* (123). This may be explained by divergent functions of SPHK isoforms in inflammation, as has been noted in a model of inflammation-induced colon cancer, where SPHK2 ablation triggered SPHK1-dependent cytokine production in myeloid cells and thus promoted M1 macrophage activation (124). The exact role of SPHK2 in macrophage activation is, however unclear. Besides promoting M1-like cytokine production, an increase in anti-inflammatory macrophages was reported in SPHK2-deficient obstructed kidneys. Treating SPHK2-deficient murine BMDM with IL-4 or IL-13 induced a more pronounced M2 profile compared to wild type macrophages (125). Thus, macrophage SPHK2 may restrict M1 as well as M2 activation. Further mechanistic investigations will be required to support this claim.

Despite its role in providing S1P as an intracellular cofactor, SPHK1 activation by inflammatory triggers may increase extracellular S1P, thereby provoking S1P receptor activation. The accompanying cell response appears to be highly context and receptor-dependent. Initial studies in human alveolar macrophages suggested that S1P alone induced NADPH-oxidase (NOX)2-dependent production of ROS (126) to promote IL-1β and TNF-α production by murine peritoneal macrophages (127). These findings were recently supported by a study suggesting that S1P stimulation of murine BMDM triggered the expression of inflammatory markers including TNF-α, CCL2, and inducible NO-synthase (iNOS), which were suppressed by targeting S1PR2/3 and downstream c-Jun N-terminal kinase (JNK) activation, again exemplifying the cooperate potential of S1PR2/3 signaling in macrophages (128). Increased iNOS expression was also observed when murine BMDM were subjected to LPS/IFN-γ treatment with the addition of exogenous S1P (82). The impact of S1PR3 on promoting inflammatory macrophage activation was substantiated in studies using murine microglia *in vitro* and a model of brain ischemia. A S1PR3 specific antagonist and siRNA-mediated depletion of S1PR3 reduced LPS-triggered expression of TNF-α, IL-6, and IL-1β (129). Moreover, LPS-induced expression of inflammatory genes such as iNOS, COX-2, IL-1β, IL-6, and TNF-α in primary peritoneal macrophages was reduced by an S1PR3 antagonist (130). S1PR3 may therefore be viewed as an inflammatory receptor in macrophages. While the majority of inflammatory macrophage markers depend on transcriptional induction, IL-1β maturation and release in response to microbial stimulation requires expression and activation of inflammasomes in macrophages, including the NLRP3 inflammasome, a protein complex consisting among others of the eponymous NLRP3 and inflammatory caspase-1 (131). S1P was shown to selectively promote the expression of NLRP3 among inflammasome components downstream of S1PR1 in tumor-associated macrophages (TAM), as well as LPS-stimulated mouse BMDM and human primary monocyte-derived macrophages (132). Besides NLRP3 expression, S1PR1 was also involved in promoting ATP release at least in a murine macrophage cell line, which is one of the triggers of NLRP3 inflammasome activation (133). Another activating mechanism of the NLRP3 inflammasome, the release of cathepsin B from lysosomes, was associated with S1PR2 signaling (134). Accordingly, levels of IL-1β and IL-18, which are inflammasome dependent, were reduced in the serum of S1PR2-deficient mice challenged with endotoxin (135). Thus, S1P via S1PR1/2, although not through converging signaling pathways, may cooperate toward NLRP3 inflammasome assembly and activation, promoting IL-1β maturation (136). Interestingly, the effect of S1PR1 on NLRP3 expression suggests that S1PR1 operates independently of canonical NF-κB or classical mitogen-activated protein kinase (MAPK) cascades triggered downstream of toll-like receptor (TLR), TNF receptor, or IL-1 receptor activation. Along these lines, antagonism of S1PR3, which generally reduced LPS-triggered inflammation in peritoneal macrophages, restricted caspase-1 but not NLRP3 expression (130). Indeed, S1P blocked LPS-dependent stimulation of NF-κB activation and downstream production of inflammatory cytokines such as TNF-α in murine and human macrophages (137–139), and to attenuate TLR2-dependent NF-κB activation in human monocytes (140). Also, S1PR1 triggered STAT3 signaling to promote induction of heme oxygenase-1 (73), as well as IL-6. Particularly, S1PR1 signaling promoted IL-6 production in a STAT3-dependent feedback loop, where IL-6 induced S1PR1 expression in mouse macrophages *in vitro*, in a model of sickle cell disease, and in dextran sodium sulfate (DSS)-induced colitis (124, 141). In RAW264.7 macrophages S1PR1 but also S1PR2 were involved in LPS and palmitate-induced IL-6 production (120). Moreover, S1PR1 signaling increased ARG1 activity in mouse macrophages to block the production of NO, which was induced by microbial stimuli in concert with IFN-γ, although the impact of STAT3 signaling in this context was not tested (137). S1P alone or in the supernatant of apoptotic tumor cells also elevated prostaglandin E_2_ (PGE_2_) production, which required stabilization of COX-2 mRNA, likely via S1PR1 (119, 142, 143). These data suggest that S1PR1 may limit microbial-induced inflammatory pathways such as canonical NF-κB activation, but induces inflammatory mediators such as IL-1, IL-6, and PGE_2_ under conditions characterized by low-grade inflammation, as found e.g., in tumors (124, 132).

The role of S1PR2 in macrophage activation appears less clear. While it was associated with inflammasome activation as outlined above, it was required for induction of ARG2 in RAW 264.7 macrophages stimulated with apoptotic cells (144), which required the transcription factor cAMP response element-binding (CREB). S1P also augmented cAMP levels in PGE_2_ or isoproterenol-stimulated RAW 264.7 macrophages through S1PR2 (145). Therefore, S1PR2 signaling is coupled to increasing cAMP levels in macrophages, which was connected to resolution of inflammation (146). Pro-resolving macrophages are also generated by the interaction with apoptotic cells, which might provide a connection between apoptotic cell-derived S1P and resolution of inflammation by establishing resolution type macrophages downstream of S1PR2. However, this hypothesis remains to be tested. The impact of S1PR4 and S1PR5, whose expression is low in most macrophages, toward macrophage polarization is largely unclear. Activation of S1PR4 primed apoptotic tumor cell-stimulated macrophages for signaling via the nerve growth factor receptor TrKA, which induced among others IL-6 and IL-10 expression (147). It is unclear if S1PR4 activation has a similar effect under other conditions. S1PR5 was so far only connected to impaired phagocytosis as outlined above.

In conclusion, SPHK activity and S1PR signaling emerge as important regulators of macrophage polarization, although the impact of some components of this machinery such as SPHK2, S1PR4, and S1PR5 needs to be tested in the future. Since macrophages are implicated in the development of inflammatory diseases, altered functional macrophage responses by the S1P system are expected to affect such conditions. This will be outline in the next chapter.

## The Eater Gone Rogue—S1P and Macrophage Function in Disease

### Cancer

Already in 1863, Rudolf Virchow observed that tumors are heavily infiltrated by leukocytes and proposed that a chronic inflammatory milieu promotes cancer initiation and progression (148). In the last decades it became clear that the immune system and its effector cells exhibit a multifaceted role in carcinogenesis. The immune system is capable of tumor rejection but paradoxically also able to promote cancer progression, which is mediated by the tumor microenvironment triggering immune tolerance (149, 150). One group of effector cells that contribute to the multifaceted role of the immune system in carcinogenesis are tumor-associated macrophages (TAM), which depending on their phenotype contribute to a pro- or anti-tumor immune response. Whereas, TAM expressing M1 markers produce pro-inflammatory cytokines and ROS that are crucial for tumor cell killing, TAM expressing M2 markers suppress an anti-tumor immune response by producing anti-inflammatory cytokines, which causes immune suppression and in the long term tumor outgrowth (151–153).

Tumor cells themselves can produce factors that activate and shape a pro-tumor M2-like TAM phenotype during tumor escape, provoking tumor progression including metastasis. One of the factors produced by tumor cells is S1P. S1P, already discussed as a pro-survival factor, exhibits pro-tumor functions by adding to tumor cell transformation, survival, migration, and neovascularization in different cancer types such as breast, colon and prostate cancer (154). We previously showed that S1P is released by apoptotic breast cancer cells and polarizes macrophages toward a M2-like phenotype, characterized by reduced TNF-α, IL-12 but increased IL-8 and IL-10 secretion (138). Tumor cell specific secretion of S1P, concomitant macrophage M2-like polarization, and subsequent tumor growth was recapitulated in a more recent study. Mrad et al. reported that inhibition of SPHK1-dependent production of S1P by B16 melanoma cells increased the number of M1-polarized macrophages and tumor growth. The latter was, however, mediated by a macrophage-independent mechanism, since tumor growth was accelerated by SPHK1/S1P-dependent production of transforming growth factor (TGF)-β by melanoma cells themselves (155). Apparently, not only the phenotype transition from inflammatory M1-like to immune-suppressive M2-like macrophages contributes to cancer progression, but also an overshooting inflammatory response mediated by macrophages may provoke cell transformation and tumor growth as shown for colitis-associated colon cancer (CAC). Thereby, SPHK/S1P/S1PR-dependent activation of macrophages and the subsequent production of pro-inflammatory cytokines were established as important factors in contributing to chronic intestinal inflammation resulting in CAC development. This has been proposed to be in part macrophage dependent, when SPHK1 expression was upregulated as a result of SPHK2 deletion, thereby enhancing S1P levels. In this setting, intracellular S1P activated NF-κB signaling, which culminated in pro-inflammatory gene transcription of IL-6 and TNF-α. Both cytokines triggered a feed-forward amplification loop with TNF-α amplifying NF-κB signaling and IL-6 maintaining persistent S1PR1-dependent STAT3 activation, supporting chronic inflammation and CAC development (124). Another study showed that blocking the SPHK/S1P axis and thus, macrophage activation, attenuates colon cancer. Mechanistically, inhibition of SPHK1 in peritoneal macrophages reduced COX-2 and TNF-α expression, which lowered the formation of aberrant crypt foci in the colons of mice injected with the carcinogen Azoxymethane (156). In terms of S1P-dependent production of pro-inflammatory cytokines by macrophages it apparently needs to be discriminated between the extra- and intracellular actions of S1P during carcinogenesis. As mentioned, extracellular S1P formation produced by apoptotic tumor cells induces the secretion of anti-inflammatory cytokines, while intracellular S1P induces a pro-inflammatory cytokine signature of macrophages in the context of cancer.

Besides affecting cytokine production, the S1P/S1PR axis can induce pro-angiogenic properties of macrophages and therefore contribute to tumor angiogenesis and, consequently, metastasis. Macrophage-specific deletion of S1PR1 in a mammary carcinoma model enhanced lung metastasis by inducing tumor lymphangiogenesis. Mechanistically, S1PR1 signaling in lymph vessel-associated macrophages induced NLRP3 expression and IL-1β production, which showed direct pro-lymphangiogenic activity, thereby accelerating tumor progression by promoting metastasis (132). Beside the lymphatics, metastasis also occurs via the bloodstream, which requires tumor angiogenesis. One major driver of tumor angiogenesis is hypoxia, which at the same time affects macrophage biology in inflammation, cancer, or infection. The oxygen-sensitive transcription factors hypoxia inducible factors 1α and 2α (HIF-1α, HIF-2α) are the master regulators toward decreased oxygen tension, coordinating many of the multiple hypoxic responses. In tumor cells there is evidence that hypoxia causes a rapid activation of SPHK1, preceding HIF-1α accumulation (157). Although details remain unknown, ROS appeared to activate SPHK1, while the accumulating S1P, via the Akt/GSK3ß pathway, attenuated HIF-1α proteasomal degradation. In renal cell carcinoma, SPHK1 activity controlled HIF-2α expression (158) and the S1PR antagonist FTY720 attenuated both HIF-1α and HIF-2α accumulation in several human cancer cell lines (159). S1P released by dying cancer cells also triggered HIF-1α accumulation in macrophages downstream of S1PR1, even under normoxic conditions (160). In contrast to hypoxic tumor cells, where S1P may be self-sufficient to accumulate HIF-1α the situation in macrophages seems different. Macrophages sensing S1P from dying cancer cells required a second stimulus, most likely transforming growth factor (TGF)-β to stabilize HIF-1α under normoxia. HIF-1α expression under these conditions established a pro-angiogenic macrophage phenotype (160), indicating that S1P/S1PR1 signaling may promote tumor angiogenesis in general.

To sum up, studies so far may indicate a critical role of the SPHK/S1P/S1PR axis in macrophage activation and polarization, contributing to cancer development and metastasis. However, more studies are needed to clarify the exact role of the S1P-induced functional consequences for macrophage biology in different tumor entities, especially in respect of utilizing the SPHK/S1P/S1PR axis for cancer therapy. As outlined above, inhibiting one SPHK will provoke over-activation of the remaining SPHK and this may cause severe side effects. Therefore, a cell-type specific or S1PR-specific inhibition of the S1P/S1PR axis appears a more rational approach for intervention. Interestingly, in contrast to S1PR1, S1PR2 was reported to limit tumor development and angiogenesis in one study, which involved S1PR2 on myeloid cells (161). Thus, S1PR1 and S1PR2 may show opposing effects on tumor angiogenesis, making S1PR1 a potentially more interesting target when focusing on TAM.

### Atherosclerosis

Macrophages are key cellular mediators of atherosclerosis, as they accumulate in atherosclerotic plaques and the macrophage content and activation state are linked to the progression and the regression of atherosclerosis (162). Macrophages in atherosclerotic plaques are exposed to mediators in the circulation, including S1P. In the circulation S1P is mainly derived from erythrocytes, platelets, or the endothelium. About two-thirds of the circulatory S1P is associated with HDL, followed by its association with albumin and other lipoproteins (163). HDL is known to limit inflammatory responses during atherogenesis. Besides, HDL is also recognized for its general host defense activity, which is linked to its ability to scavenge and limit endotoxin toxicity as well as immune cell modulatory responses, affecting the cholesterol content in plasma membrane lipid rafts (164). HDL functions as a reservoir for several proteins and lipids with immunomodulatory activities, among them S1P. ApoM, a genetic variant of ApoA-1, is mainly associated with HDL and the carrier of S1P in HDL (165). A lipophilic pocket of ApoM not only ligates S1P but also molecules such as oxidized phospholipids or retinol, suggesting some kind of competition (166). ApoM levels are subjected to variations, with drastically reduced amounts during diseases such as atherosclerosis, coronary artery disease, or myocardial infarction as well as acute phase responses. Several studies attributed individual HDL functions as partially or entirely dependent on HDL-bound S1P (167). The mechanism for ApoM-mediated modulation of S1P function may reside in retarding S1P degradation (168) and/or strengthening its agonistic properties by binding HDL via scavenger receptors and thereby bringing S1P in close proximity with S1PRs (169).

The nature of S1P in the pathogenesis of atherosclerosis is ambivalent, although concepts on a defensive role prevail. Toward a protective function, S1P is supposed to promote survival and prevent apoptosis of endothelial cells and macrophages, to induce phosphorylation of endothelial-type NO-synthase (eNOS), which provokes vessel relaxation, to preserve endothelial barrier function by stabilizing cell-cell junctions, and to attenuate attachment of blood cells to the endothelium by inhibiting expression of endothelial cell adhesion molecules (170, 171). Harmful properties of S1P are discussed concerning its ability to recruit lymphocytes to sites of inflammation, to act chemotactic and stimulatory for other immune cells, i.e., monocytes/macrophages, to indirectly shape the atheroprotective B1-cell population, or to augment thrombin-induced expression of tissue factor in endothelial cells to foster the coagulation cascade (170, 171). Concerning macrophages, S1PR2 retains them in atherosclerotic plaques and regulates their inflammatory cytokine secretion to promote atherosclerosis (135). Also S1PR3 on monocytes/macrophages contributes to their accumulation in atherosclerotic lesions, thereby adding to a pro-inflammatory, pro-atherogenic environment (172). Hereby S1PR2 and S1PR3 signaling both promote macrophage accumulation in plaques, although by exerting opposing effects on monocyte/macrophage migration. However, other studies see the macrophage-S1P-axis in an atheroprotective context. S1P and its analog FTY720 reduced atherosclerotic lesions, both in the aortic root and brachiocephalic artery, and almost completely blunted necrotic core formation (173). Although a direct connection to macrophages in atherosclerotic plaques was not made, this observation may refer to the modulating role of S1PR signaling in macrophage recruitment, since FTY20 targets S1PR3 but not S1PR2. Alternatively, it may be connected to polarization of macrophages toward an anti-inflammatory, regenerative, healing phenotype as discussed above. In line, providing KRP-203, an S1PR1 agonist, to low-density lipoprotein receptor-deficient mice on a cholesterol-rich diet reduced atherosclerotic lesion formation and reduced macrophage pro-inflammatory activation (174). These experiments would argue for S1PR1 in mediating the anti-atherogenic effects of S1P. Besides the ability of S1P to induce alternative macrophage polarization and thereby to attenuate oxidized LDL-induced lipid accumulation, the atheroprotective effect of S1P was also related to its ability to enhance cell survival and to attenuate macrophage pro-apoptotic signaling (175). Mechanistically, HDL-associated S1P attenuated macrophage apoptosis by activating STAT3 and causing survivin expression, presumably via cooperate signaling through S1PR2/3, as recapitulated by pharmacological interventions (75). As many HDL effects are attributed to its S1P load, it is of interest that the physiologically crucial and most relevant role of HDL in reverse cholesterol transport is now also proven to be affected by S1P (176). The transcriptional and functional ABCA1 regulatory pathway, facilitating cholesterol efflux, demanded S1PR3. The authors established LXR to be involved in S1P facilitated cholesterol efflux and identified the critical role of S1PR3.

It is interesting to note that approaches are undertaken to make use of the beneficial HDL-S1P signaling axis for the treatment of diseases. Only HDL, manufactured to incorporate S1P, was cardioprotective in a model of ischemia reperfusion injury (177) and S1P-loaded HDL enhanced eNOS activation in endothelial cells (178). In general, the anti-inflammatory HDL function can be boosted by S1P-loading and exploited by S1P receptor-targeting to prevent and even turn off ongoing inflammation (179). Another strategy follows the observation that ApoM levels are correlative to biological S1P-signaling. Resveratrol, a proposed supplement to prevent atherosclerosis, is reported to modulate S1P levels by affecting ApoM levels (180). It can be speculated that some of the reported anti-atherosclerotic effects of resveratrol can be explained by increasing plasma levels of ApoM in conjunction with its S1P-association.

Although many details on the role of S1P and macrophages during atherosclerosis still need to be discovered, a gross simplification would favor anti-atherosclerotic actions of S1PR1 and S1PR3, while pro-atherosclerotic functions of S1PR2 may dominate. Mechanistically, the anti-inflammatory impact of S1P toward macrophages, likely transmitted via S1PR1, may add to convey atheroprotective signals. Uncertainties remain, as we are not aware how S1P concentrations, either HDL-bound or associated with other carriers, develop over time with plague progression and how the S1P receptor profile may change in early vs. late stages of the disease. As macrophages are prone to many environmental incoming signals, GPCR activation by S1P may dominate, be modulated, or be overruled. This makes predictions on the macrophage S1P-S1PR signaling axis difficult. However, controlled *in vivo* experimentation, using genetically modified animals in combination with pharmacological tools that are progressing toward higher selectivity, will help to answer some of the demanding questions in the future.

### Fibrosis

Macrophages are one of the key players during resolution of inflammation as the wound healing response has to be tightly regulated (181, 182). Disturbances within any stage of the wound healing process may cause chronicity, while an overshooting healing response can induce fibrosis within different organs such as lung, liver, heart, or kidney. Tissue fibrosis is characterized by increased proliferation and activation of fibroblasts that trigger excessive accumulation of extracellular matrix components eventually initiating organ failure and death (182, 183). Macrophages may serve as critical mediator during fibroblast activation and proliferation by releasing pro-fibrotic mediators such as TGF-ß, IL-13, or platelet-derived growth factor (PDGF) (184). There is evidence that the infiltration of anti-inflammatory M2 macrophages into fibrotic areas of the lung is a key regulator for the development and progression of idiopathic pulmonary fibrosis (IPF) (185, 186).

A role for S1P in IPF progression was already assumed by showing that serum and bronchoalveolar lavage of diseased mice or patients exhibit increased S1P levels and show enhanced SPHK1 protein expression, both correlated with impaired lung function (187, 188). Mechanistically, S1P is implicated in secreting pro-fibrotic factors that cause the excessive activation and proliferation of fibroblasts, thereby advancing tissue fibrosis. Specifically, studies pointed to a role of the SPHK/S1P/S1PR axis in TGF-ß-driven fibrosis induction. This was demonstrated by blocking SPHK1, which in turn reduced TGF-ß secretion and lung fibrosis in murine models of IPF (187, 189). It has also been shown that the S1P/S1PR axis in macrophages contributes to the production of pro-fibrotic factors and thereby adds to IPF development (190). Along those lines, Zhao et al. used S1PR2^−/−^ mice and noticed attenuated IPF in animals subjected to bleomycin. In this model S1PR2-expressing alveolar macrophages most likely promote IPF as shown by bone marrow transfer experiments and the enhanced S1PR2-dependent production of pro-fibrotic IL-13 that initiates a STAT6-dependent response in macrophages. More mechanistic studies using macrophage-specific S1PR^−/−^ mice will be needed to decipher the exact role of the S1P/S1PR axis in the development and progression of IPF. The SPHK/S1P/S1PR signaling axis also accelerates liver fibrosis by directly activating fibroblast motility and fibrosis-induced angiogenesis (191, 192). In a more recent study it became apparent that SPHK1-induced CCL2 secretion from Kupffer cells activated fibroblasts and thereby fostered progression of liver fibrosis (193). The finding that S1P signaling adds to the pathogenesis of tissue fibrosis was already shown in the kidney, when partial nephrectomized rats were treated with FTY720. Blocking the S1P/S1PR axis by FTY720 diminished renal fibrosis, characterized by reduced expression of TNF-α, TGF-β, and the production of extracellular matrix proteins (194). For renal macrophages protection toward fibrosis was linked to SPHK2-dependent S1P signaling. SPHK2-deficient kidney-resident macrophages shifted toward the M2 phenotype due to changes in the glycolytic pathway, which reduced renal fibrosis by lowering the production of pro-inflammatory cytokines such as IL-1β and TNF-α (125). Evidently, S1P contributes to macrophage-dependent fibrotic responses by shaping their activation, particularly the release of pro-fibrotic cytokines/chemokines such as TGF-ß, IL-13, or CCL2. Addressing the distinct role of S1P in macrophage-driven fibrosis in detail may open the potential to foster mechanisms toward resolution of fibrosis.

### I/R Injury

Ischemia-reperfusion-induced injury (I/R injury) plays a major role during stroke and myocardial infarction, and is accompanied by inflammation that promotes injury. Macrophages play a crucial role in resolving inflammation and promoting repair following ischemic injury (195–197). Targeting S1P receptors has shown promising results in I/R injury models. However, while macrophages are used as read-out parameters, their functional involvement largely remains unclear. S1P levels during resolution of focal cerebral ischemia in mice increased (198), when macrophages promote repair. Treating mice in a model of experimental stroke with FTY720 reduced lesion size and improved neurological function, which was accompanied by decreased numbers of activated microglia/macrophages in the ischemic lesion (199). Reduced inflammatory microglia/macrophage infiltration was confirmed in models of focal cerebral ischemia and observed under long-term protective effects of FTY720 (200). Whether reduced infiltration of inflammatory microglia/macrophage were a result of reduced recruitment into the affected area or due to changes in cell activation remains an open question. Intraocular injection of a humanized monoclonal S1P antibody (sonepcizumab) into ischemic retina significantly reduced the macrophage influx in oxygen-induced ischemic retinopathy (201). Attenuated macrophage infiltrates and their proinflammatory cytokine expression were furthermore observed when applying the S1PR1 agonist SEW2871 in mice subjected to hepatic (202) or renal I/R injury (203). Based on the observed lymphopenia in these models, SEW2871 acted as a functional S1PR1 antagonist as expected. However, lymphopenia was not the reason for protection as mice, harboring a selective S1PR1 knockout in proximal tubule cells, were protected as well (204). In some analogy, in a model of cisplatin-induced nephropathy performed in mice with a deletion of S1PR1 in tubule cells reduced kidney damage and a lower level of proinflammatory cytokines and infiltrated macrophage were noticed (205). To conclude, the protective effect of S1PR agonism in the kidney was largely macrophage independent, whereas tissue regeneration following I/R injury required S1PR1/3 signaling and was linked to the release of neutrophil gelatinase-associated lipocalin (LCN2) from macrophages, again underscoring the role of macrophages in tissue regeneration after I/R injury (206). S1PR1/3 activation also protected against cardiac ischemia-reperfusion injury as the accompanying tissue injury in mice was reduced by myonectin, which triggered S1P and protected cardiomyocytes from apoptosis and macrophages from their inflammatory activation. Administration of the S1PR1/3 antagonist VPC23019 reduced the protective potential of myonectin and increased myocardial injury (207). Understanding the role of macrophages in S1P-dependent protection from I/R injury will require further studies by e.g., employing macrophage-specific S1PR^−/−^ mice.

### Infection

Sphingolipids are involved in immunity to infection, with prominent roles being assigned to ceramide and sphingosine (208–210). However, an impact of the SPHK/S1P/S1PR axis under infectious conditions requires further studying. Data from cell culture suggest a role of S1P in pathogen uptake and killing, while information from *in vivo* models is scarce. SPHK1 was required to form lung granuloma and prevented brain infection with a particular *C. neoformans* strain that is restricted to intracellular replication in macrophages (108). Logically, SPHK1-deficient showed a higher susceptibility to *C. neoformans* infection (108). SPHK1 was also required for the protective principle of glucocorticoids in a model of acute lung injury, triggered by LPS and oleic acid. Downstream of the glucocorticoid receptor SPHK1 was upregulated in macrophages, provoking a systemic S1P increases and reducing inflammatory cell infiltrates (211). Enhanced SPHK1 expression was also observed in macrophages in inflamed murine and human lungs in pneumonia, while genetic SPHK1 deletion protected mice from pneumonia-induced hyperpermeability. Unfortunately, the role of macrophage-specific SPHK1 in this process remained unclear (212). SPHK2-deficient mice showed a higher susceptibility to *Streptococcus pneumonia* induced lung inflammation, although there was no change in neutrophil function, leaving room for a role of macrophages (213).

With regard to S1P receptors, deletion of S1PR2 was protective in models of bacterial sepsis. S1PR2 promoted macrophage pyroptosis, which is linked to a cytokine storm, upon *E. coli* infection, while a S1PR2 knockout improved survival (76). Survival of S1PR2 knockout mice was also seen during cecal ligation and puncture or intratracheal administration *E. coli*, which was linked to an enhanced phagocytic function of S1PR2-deficient macrophages (98). S1PR3 supports ROS generation in macrophages, thereby aiding in killing bacteria and promoting phagosome maturation upon cecal ligation and puncture, where S1PR3 knockout showed increased lethality (104). Septic patients with monocytes showing enhanced S1PR3 expression cleared bacteria more efficiently, which was linked to a preferable outcome (104). Accordingly, the S1PR agonist FTY720 prevented clearance of bacteria albeit increasing colonic inflammation and neutrophil infiltration in a model of gastrointestinal infection with the mouse enteric pathogen *Citrobacter rodentium*. FTY720 targets all S1P receptors, with the exception of S1PR2, showing short term agonistic activity, followed by receptor desensitization due to their degradation. Thereby FTY720 traps lymphocytes in secondary lymphatic organs by disabling them to follow the S1P gradient toward the circulation. FTY720 treated animals therefore exhibit peripheral blood lymphopenia with significantly lower numbers of colonic dendritic cells, macrophages, and T cells. Infected mice treated with FTY720 revealed an impaired innate immune response and reduced type 1 adaptive immunity (214). Therefore, targeting S1PRs with rather non-specific tools will likely not be beneficial during infection. However, it might be worth considering to selectively targeting S1PR2 as it likely increases the anti-microbial macrophage function and enhances macrophage survival. Moreover, S1PR2 antagonism will probably not induce immune paralysis, since S1PR1 is the important receptor promoting lymphocyte recruitment to the circulation.

## Conclusions

**Figure 2:**
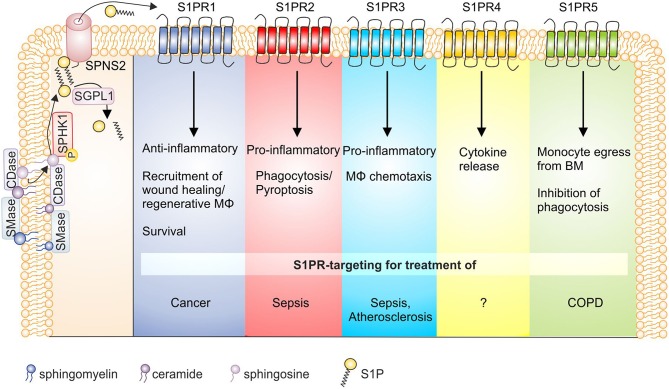
S1P receptor signaling and macrophage function in disease. S1P is generated by the sequential breakdown of sphingomyelin and ceramide to sphingosine by sphingomyelinases and ceramidases, respectively, and the subsequent phosphorylation of sphingosine by sphingosine kinases. S1P is then degraded by S1P lyase or secreted from cells via the transporter SPNS2. Activation of S1P receptors on macrophages then triggers functional responses as indicated, whose targeting might be of interest in disease settings. Details can be found in the main text. BM, bone marrow; CDase, ceramidase; COPD, chronic obstructive pulmonary disease; MΦ, macrophage; S1P, sphingosine-1-phosphate; S1PR, sphingosine-1-phosphate receptor; SGPL1, S1P lyase; SMase, sphingomyelinase; SPHK, sphingosine kinase; SPNS2, Spinster homolog 2.

Macrophages are key players in maintaining tissue homeostasis, which requires a remarkable repertoire to sense microenvironmental cues that signal disturbed homeostasis. S1P is a good example of a sensing/signaling molecule, since its tissue levels, except for blood and lymph, are constitutively low. Rising S1P levels therefore imply an altered microenvironment, which is sensed by macrophages and their progenitors. This provokes monocyte/macrophage trafficking, survival, and altered effector functions. These changes are involved in a number of diseases as highlighted in this review (Figure 2). The source of S1P and the receptor profile are critical determinants of the S1P impact on macrophages. Intracellular S1P produced by **SPHK1** mostly promotes inflammatory macrophage activation and increases anti-microbial properties including phagosome maturation.

S1P signaling through individual S1PRs has pleiotropic and sometimes even divergent effects. In monocytes/macrophages **S1PR1** recruits wound healing and/or regenerative macrophages (215), acts as a survival signal (88) and predominantly causes anti-inflammatory macrophage polarization (137). In IL-4 stimulated macrophages S1PR1 expression is enhanced (81), while in pro-inflammatory activated phagocytes S1PR1 and S1PR4 appeared downregulated, at least at mRNA level (82). Moreover, S1PR1 stimulated EPO-signaling in macrophages to enhance apoptotic cell clearance through PPARγ, which adds to the anti-inflammatory/regenerative macrophage phenotype (90). However, when S1PR1 is activated in the context of endogenous SPHK1 activation in macrophages, pro-inflammatory effects may prevail (124). Therefore, targeting S1PR1 affects inflammatory macrophage activation needs to be approached in a context-dependent manner, although it emerges as a promising target in cancer. **S1PR2** generally seems to oppose S1PR1 signals. The receptor regulates macrophage retention in atherosclerotic plaques and provokes cytokine secretion to promote inflammation (135). However, more recent data proposed a S1PR2-G_12/13_ signaling axis in macrophages that augmented protective B-cell populations to ameliorate atherosclerosis (216). This receptor also enhanced Fcγ receptor-facilitated phagocytosis in response to antibody opsonized particles, but not complement-mediated phagocytosis (97). It was also proposed that S1PR2 impaired phagocytosis and antimicrobial defense in the pathogenesis of sepsis (98). Furthermore, S1PR2 deficiency/S1PR2 inhibition decreased macrophage pyroptosis and improved survival in *E. coli* sepsis, posing this receptor as a promising therapeutic approach during sepsis (76). These observations support the notion of S1PR2 in contributing toward proinflammatory macrophage polarization (128), although it may play a role in inflammation resolution by increasing cAMP levels as well (144). **S1PR3** mediates chemotaxis of macrophages, *in vitro*, and provokes migration of cells to plaques in atherosclerotic mice (172) and recruits macrophages in bile duct-ligated mice to promote hepatic inflammation and fibrosis (85). In general, S1PR3 appears to mediate pro-inflammatory responses. This is coupled to antimicrobial function as S1PR3 expression was elevated in septic patients, linked to bacterial clearance and a better outcome (104). Mechanistically, bacterial killing in macrophages was fostered by enhanced ROS formation and phagosome maturation. Activating S1PR3 on macrophages therefore might be beneficial to promote antimicrobial immunity. **S1PR4** is less well studied than S1PR1 to S1PR3, but is abundant on immune cells, among them macrophages (217). S1PR4 was required to produce the Th17 polarizing cytokine IL-6 by dendritic cells (218). This may also be true for macrophages since activation of this receptor on TAM by S1P, released from apoptotic cells, caused formation of tumor promoting cytokines, including IL-6 and IL-10 (147). Under pro-inflammatory conditions macrophages downregulate S1PR4 (82), which has also been shown for plasmacytoid dendritic cells (219). Whether S1PR4 downregulation is required for pro-inflammatory signaling to take place will require further studying. There is evidence that **S1PR5** is involved in the egress of patrolling monocytes from the bone marrow (220), thereby potentially contributing to the tissue macrophage pool during inflammation. As S1P did not function as a chemoattractant for these cells nor did it affect their viability *in vitro*, detailed mechanisms remained unexplored. Alveolar macrophages from patients with COPD are defective in their ability to phagocytose apoptotic cells. As a significant association was noted between S1PR5 expression and both lung function as well as defective phagocytosis it is concluded that this receptor might be a potential therapeutic target in COPD (93).

Besides evidence suggesting a role of S1P/S1PR signaling in shaping macrophage-specific tissue homeostasis, a number of questions still need to be addressed. The role of S1P in emerging areas such as macrophage immune metabolism and innate memory formation may require our attention. This is supported by findings that **SPHK2** deficiency reduces glycolysis in macrophages (125), and produces intracellular S1P as a cofactor for HDACs to modulate epigenetics (221), both of which are hallmarks of trained macrophage immunity (222). Moreover, experiments using specific deletion of individual components of this signaling axis, particularly individual S1PRs in macrophages will allow identifying potential pharmaceutical targets to be exploited for disease conditions mentioned above. This is of special importance given the sometimes antithetic properties of signaling through individual S1PRs. Targeting the SphK/S1P/S1PR axis is already the object of a number of clinical trials. Most notably S1PR1 modulators (Fingolimod, MT-1303, Ozanimod) are tested or already in clinical use for treating inflammation-driven diseases such as multiple sclerosis, inflammatory bowel disease and psoriasis (223). These drugs are thought to act mainly through the induction of sustained lymphopenia by trapping T cells in lymphatic organs. Thereby, S1PR1 modulators dampen the inflammatory response and thus reduce disease severity. It is unclear how these drugs affect macrophage biology in patients. Targeting macrophage S1PR1 in cancer may be of interest, as outlined above. However, in the case of cancer therapy, as well as during infection, sustained lymphopenia triggered by S1PR1 antagonism may rather be disadvantageous, since T cells are needed at the tumor site for a proper anti-tumor response (224). Targeting S1P levels globally using the anti-S1P monoclonal antibody Sonepcizumab failed in a phase II study of metastatic renal cell carcinoma (225). A phase I clinical trial with Safingol, which inhibits SphK besides other kinases, published in 2011 showed potential in treating solid tumors when combined with chemotherapy (226). Administration of Safingol alone was ineffective and further clinical were so far not conducted. The SPHK2 inhibitor ABC294640 showed promise in a phase I trial in patients with advanced solid tumors (227). However, given the opposing effects of signaling through individual S1PRs, targeting individual S1PRs may be more rational to unleash the full potential of S1P modulators. In the context of cancer and with a focus on immunity, S1PR4 may represent an interesting drug target since it is mainly expressed on immune cells including macrophages and T cells, and does not affect T cell trafficking similar to S1PR1.

## Author Contributions

All authors listed have made a substantial, direct and intellectual contribution to the work, and approved it for publication.

### Conflict of Interest Statement

The authors declare that the research was conducted in the absence of any commercial or financial relationships that could be construed as a potential conflict of interest.
